# Transcriptome Profiling of Sexual Maturation and Mating in the Mediterranean Fruit Fly, *Ceratitis capitata*

**DOI:** 10.1371/journal.pone.0030857

**Published:** 2012-01-27

**Authors:** Ludvik M. Gomulski, George Dimopoulos, Zhiyong Xi, Francesca Scolari, Paolo Gabrieli, Paolo Siciliano, Anthony R. Clarke, Anna R. Malacrida, Giuliano Gasperi

**Affiliations:** 1 Department of Animal Biology, University of Pavia, Pavia, Italy; 2 W. Harry Feinstone Department of Molecular Microbiology and Immunology, Bloomberg School of Public Health, Johns Hopkins University, Baltimore, Maryland, United States of America; 3 Discipline of Biogeosciences, Queensland University of Technology, Brisbane, Queensland, Australia; The Centre for Research and Technology, Hellas, Greece

## Abstract

Sexual maturation and mating in insects are generally accompanied by major physiological and behavioural changes. Many of these changes are related to the need to locate a mate and subsequently, in the case of females, to switch from mate searching to oviposition behaviour. The prodigious reproductive capacity of the Mediterranean fruit fly, *Ceratitis capitata*, is one of the factors that has led to its success as an invasive pest species. To identify the molecular changes related to maturation and mating status in male and female medfly, a microarray-based gene expression approach was used to compare the head transcriptomes of sexually immature, mature virgin, and mated individuals. Attention was focused on the changes in abundance of transcripts related to reproduction, behaviour, sensory perception of chemical stimulus, and immune system processes. Broad transcriptional changes were recorded during female maturation, while post-mating transcriptional changes in females were, by contrast, modest. In male medfly, transcriptional changes were consistent both during maturation and as a consequence of mating. Of particular note was the lack of the mating-induced immune responses that have been recorded for *Drosophila melanogaster*, that may be due to the different reproductive strategies of these species. This study, in addition to increasing our understanding of the molecular machinery behind maturation and mating in the medfly, has identified important gene targets that might be useful in the future management of this pest.

## Introduction

*Ceratitis capitata* (Wiedemann) (Mediterranean fruit fly, medfly), is a highly invasive agricultural pest insect that has spread from its native range in East Africa and has now attained an almost worldwide distribution [1]. Medfly lays its eggs into immature host fruit, where the larvae subsequently feed causing crop loss [2]. The fly's ability to readily adapt to new environments, to complete multiple generations each year utilising different host plants as they become available, and its high reproductive capacity are all factors influencing its pest status and invasive potential [3].

The reproductive biology of medfly has been well studied at the physiological and behavioural levels [4]–[6]. Medflies emerge from the pupal stage as sexually immature males and females, but mature rapidly and can mate within two to three days of age [7]. In nature, the mating system is based on arboreal aggregations (leks) of pheromone-emitting males, which attract mature receptive females that subsequently select a mate from males within the lek [8]. Despite the extensive literature on medfly reproductive behaviour, little progress has been made towards understanding medfly mating behaviour at the molecular level, partially due to the lack of genomic information for this species. As one of the world's major horticultural pests, knowledge of the molecular machinery associated with its reproductive behaviour may help interpret its invasive success and has the potential to lead to longer-term applied benefits for the improvement of control methods such as the currently used Sterile Insect Technique [9].

The molecular basis of reproductive behaviour has been studied in the model insects *Drosophila melanogaster*, *Apis mellifera* and *Anopheles gambiae* [10]–[19]. Females of these species undergo major physiological and behavioural changes during sexual maturation, and again after insemination. The mating-induced modifications are largely the result of changes in gene expression in the brain, fat body and lower reproductive tract [16]. It has been shown that different regions of the *Drosophila* brain are responsible for sexual behaviours and several sex-differentiation factors are expressed therein [20]–[22]. Post-mating changes in insects often include egg production, oviposition, increased feeding behaviour and reduced readiness to mate. In the medfly, mating does not appear to increase egg production [23], but does induce sexual refraction in the majority of females [24]–[28]. Mating-induced behavioural changes in insects are often induced by substances within the seminal fluid that are transferred together with the sperm during copulation [29]. Although the medfly equivalents of male accessory gland proteins such as the *Drosophila* Sex peptide (Acp70A) have not yet been identified, they have been implicated in female sexual refraction and the switch in the female olfactory preference from male pheromones to fruit odours [30]–[32]. Uncharacterised ∼30 kDa proteins derived from the male accessory gland proteins have been shown to be transferred to the medfly female during copulation [33]. In another tephritid species, *Bactrocera tryoni*, radioactively labelled products of the male accessory glands were shown to be transferred during copulation and to migrate throughout the body of the female, including the head, within a few hours after mating [34]. In *D. melanogaster* large amounts of male accessory gland proteins bind to the female suboesophageal ganglion and the base of the antennal nerves [35], suggesting that their effects could be the result of interaction with the female nervous system.

Using previously identified *C. capitata* transcripts [36], we designed a high-density oligonucleotide microarray and used it to characterize gene transcript abundance in the heads of male and female adult medfly at different physiological stages. We chose the head, which contains both the brain and fat bodies, as the focus of our study. For each sex, comparisons of transcript abundance were made between immature and mature virgin individuals, and between mature virgin and mated individuals. The first of these comparisons enabled us to identify transcripts that undergo changes in abundance in the passage from the immature to the sexually mature state, while the second comparison enabled the identification of genes that change their transcript abundance in response to mating in individuals of the same age. In the analysis, particular attention was focused on genes associated with gene ontology (GO) categories related to reproduction, behaviour, chemoreception and immune system processes.

## Materials and Methods

### General approach

The experimental approach followed in this paper covered the following phases: (i) creation of the microarray; (ii) selection of flies; (iii) completion of microarray assays; (iv) data analysis; (v) validation of the transcript abundance data derived from the microarray analysis using a real-time quantitative PCR; and (vi) further analysis of immunity-related genes expressed in both the head and abdomen of immature, mature and mated males and females.

### Microarray construction

A total of 11885 assembled sequences derived from 21,253 expressed sequence tags (ESTs) from two medfly cDNA libraries from male and female heads (0–8 days old) and from 0–36 h embryos [36], together with 59 *C. capitata* sequences obtained from GenBank, formed the basis of the custom microarray. Assuming that the medfly possesses a similar number of genes as *D. melanogaster*, these sequences could represent about 80% of the medfly transcriptome. Up to two independent unique 60-mer oligonucleotides were designed for each sequence using the Array designer software (Premier Biosoft). Oligonucleotides were designed to be complementary to the 3′ end of each sequence where feasible. Microarrays were constructed by Agilent Technologies Sure Print Technology on glass slides.

### Medfly strains and RNA preparation

The ISPRA strain of *C. capitata*, established in Pavia in 1979 from a colony started in 1968 at the European Community Joint Research Centre, Ispra, Italy, with wild flies from Sicily and Greece, was used in this study. Standard rearing methods were used [37]; this included a 12 hr light-on/light-off regime with lights-on at 08:00hrs. Newly emergent adults were collected over a four hour period following lights-on and were sexed within five hours of emergence: the sexes were subsequently maintained in separate cages. As male and female medfly require two to three days to reach sexual maturity under standard rearing conditions [7], one day old (1 d) individuals were considered immature and four day old (4 d) individuals sexually mature. Twenty-four and 96 hours after emergence, the virgin male and female flies were chilled briefly and decapitated with a sterile scalpel, generating the virgin immature and virgin mature samples respectively. RNA was extracted from groups of 15 heads of each sex and age class using the RNeasy mini kit (Qiagen). RNA samples were treated to remove any contaminating DNA with the DNA-free kit (Ambion). The spermathecae were isolated from the carcases of the females and checked to confirm the absence of sperm.

To obtain mated flies, approximately 100 three day old virgin flies of each sex were introduced into a 30 cm^3^ cage shortly after lights-on. Mating in our laboratory strain of *C. capitata* begins soon after lights-on. As copulating pairs were observed they were isolated and removed from the cage in small vials. Only those pairs that remained *in copula* for at least 100 minutes were used to avoid false matings, i.e. those with little or no sperm transfer [38]. Copulation was allowed to terminate naturally and the flies were placed in separate cages according to sex. RNA was extracted from mated males and females the following day (i.e. at four days of age) as for the virgin flies. Tissue collections and RNA extractions were performed at the same time after lights-on for all the samples.

### Microarray assays

Total RNA (3 µg) was used to synthesize double-stranded cDNA using an oligo d(T)-T7 promoter primer. Complementary RNA (cRNA) probes labelled with Cy-3-dUTP and Cy-5-dUTP fluorescent nucleotides were synthesized from this double-stranded cDNA using the Agilent Technologies low-input linear amplification kit. Unincorporated fluorescent nucleotides were removed using the Qiagen RNeasy kit (Qiagen) and probe quality determined spectrophotometrically.

The experiments performed and the labelling of the cRNA probes were: 1 d virgin female head (Cy-5) against 4 d virgin female head (Cy-3); 1 d virgin male head (Cy-5) against 4 d virgin male head (Cy-3); 4 d mated female head (Cy-5) against 4 d virgin female head (Cy-3); 4 d mated male head (Cy-5) against 4 d virgin male head (Cy-3). Three biological replicate assays were performed for each experiment.

Hybridisations were performed over-night at 60°C according to the Agilent Technologies *in situ* hybridization kit. After washes the microarrays were dried using pressurised air. Microarrays were scanned using an Axon GenePix 4200AL scanner using a 10 µm pixel size and adjusting the PMT to maximise effective dynamic range. Spot size, location and quality were assessed using the GenePix Pro 6.0 software (Axon Instruments) and hybridization artefacts and damaged spots were manually flagged for removal from subsequent analyses. The dataset was filtered using the TIGR MIDAS 2.19 software [39] using a signal cut-off of 100 fluorescence units to remove low intensity spots from the analysis. The background-subtracted median fluorescence signals were normalised to eliminate dye-specific biases using the Loc-Fit method (LOWESS) in TIGR MIDAS 2.19 and in-slide duplicates were averaged using the GEPAS microarray pre-processing software [40].

### Data analysis

The normalised Cy-5/Cy-3 ratios from replicate assays were used as input for BAGEL (Bayesian Analysis of Gene Expression Levels) [41]. BAGEL infers relative transcript abundance using a Bayesian probability model. BAGEL calculates an estimated mean and 97.5% credible intervals of the relative transcript abundance of each gene in each treatment. In order to determine whether a transcript significantly changed in abundance two criteria were used, both of which had to be met: 1) a conservative criterion of non-overlapping 97.5% credible intervals for each gene in each pair of treatments, and 2) the relative abundance ratio was at least 1.74 fold (representing at least 74% higher or lower transcript abundance). The combination of these two criteria identifies high-confidence gene sets with biologically meaningful changes in transcript abundance [42]. Within BAGEL, the following pair-wise statistical comparisons were performed: 1VF>4VF, 1VF<4VF, 4MF>4VF, 4MF<4VF, 1VM>4VM, 1VM<4VM, 4MM>4VM, and 4MM<4VM (where 1VF, 4VF and 4MF represent 1 day virgin, 4 day virgin and 4 day mated females, respectively and 1VM, 4VM and 4MM represent 1 day virgin, 4 day virgin and 4 day mated males, respectively).

While the putative function (where known) of ESTs used in the microarray had been previously determined [36], the putative functions of all transcripts that changes in abundance were again checked in BLASTX searches based on their sequence similarities with *D. melanogaster* transcripts. Gene Ontology (GO) analyses were performed using GO Slimmer [43]. GO Term Enrichment analysis was performed using GOEAST [44] for customized arrays with default parameters (hypergeometric statistical test method, Yekutieli false discovery rate (FDR) under dependency, Significance level of enrichment = 0.05). The expression profiles of *Drosophila* genes were obtained from FlyMine [45]. The microarray design and assay results have been deposited in the Gene Expression Omnibus (GEO) with accession number GSE19614 in compliance with MIAME guidelines.

### Validation of transcriptome data with real-time quantitative PCR

To validate the transcript abundance data derived from the microarray analysis, real-time quantitative PCR (qRT-PCR) was independently performed on 12 genes (*takeout*, *smell-impaired 35A*, *male-specific serum polypeptide*-γ*1*, *Defensin, Relish, Ptp61F*, *PGRP-LC* and five odorant binding protein genes *Obp69a*, two *Obp19d* homologues (HS3757 and HC2265), *Obp83a* and *Obp28a* using cDNA from immature, mature virgin and mated male and female heads.

Three reference genes, *GAPDH2*, *G6PDH* and *RpL13A*, were used for relative quantification normalization [46]. The primers were designed using Beacon Designer 7 (Premier Biosoft International).

The quantity and quality of DNase-treated RNA was assessed using a Nanodrop ND-1000 spectrophotometer (Nanodrop Technologies Inc., Wilmington, DE, USA). Synthesis of cDNA was performed using 250 ng RNA in 20 µl reaction volumes using a mixture of oligo (dT) and random hexamer primers (Bio-Rad). Real-time quantification was performed using the iQ SYBR Green Supermix kit (Bio-Rad) and MiniOpticon (Bio-Rad). Cycling conditions involved an initial 95°C for three minutes, 40 cycles of 10 seconds at 95°C, 30 seconds at 57°C and 30 seconds at 68°C. A fluorescence reading was made at the end of each extension step. Three replicates were performed and specificity of the amplification products was assessed by melt-curve analysis. PCR efficiencies were above 88% for all primer pairs (Table S1). Statistical comparison of microarray and qRT-PCR (log_2_ ratio) datasets was performed using Pearson correlation analysis.

To confirm the influence of maturation and mating status on the expression of immune response related genes, qRT-PCR assays were performed using two previously identified medfly immune response genes, *Cecropin 1* and *Ceratotoxin A* [47], [48] and four transcripts that shared significant similarities with *Drosophila* genes involved in the immune response (*Relish*, *Defensin*, *Attacin A* and *PGRP-LC*) on the heads and abdomens of virgin and mated males and females. Three replicate assays were performed for each experiment and differences in the relative transcript abundance of each gene in 1 d flies and mated 4 d flies for both sexes were separately compared against transcript abundance in 4 d virgin flies of the same sex using 2-tailed t-tests.

## Results

We analysed a total of 11,885 transcripts, of which 811 displayed changes in transcript abundance during female maturation and 301 during male maturation. Transcripts that showed changes in abundance related to mating amounted to 264 in males and only 32 in females.

### Transcriptional changes during female maturation

**Figure 1:**
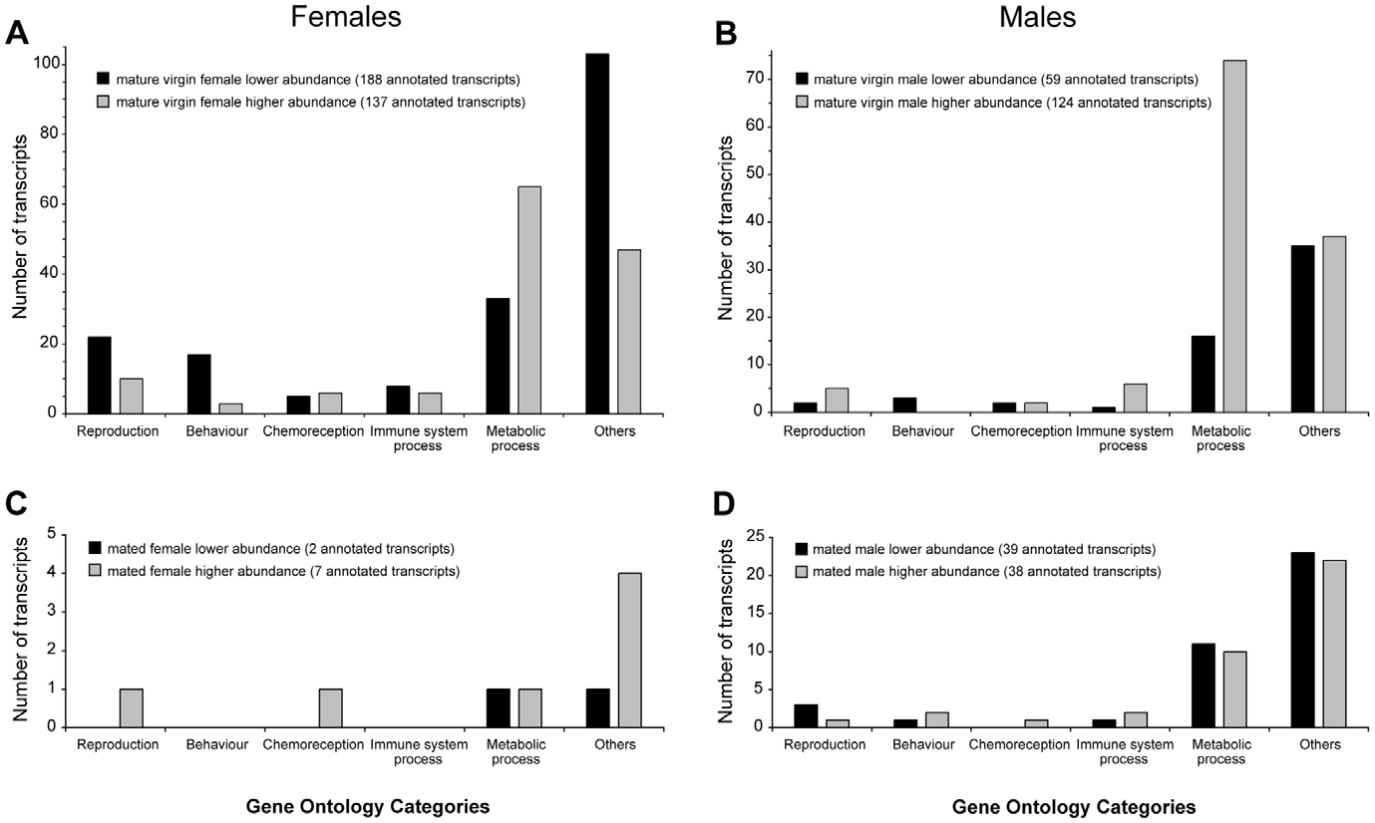
Changes in transcript abundances with maturation and mating in medfly heads. The graphs include only GO annotated transcripts and show the number of enriched transcripts in the different samples belonging to different biological categories in A. mature virgin females compared to immature females, B. mature virgin males, compared to immature males, C. mated females compared to mature virgin females, and D. mated males compared to mature virgin males.

Out of 811 transcripts (6.8%) that displayed significant transcriptional changes between immature and mature virgin female heads, almost half (49%) did not share any significant similarity with known genes. As many as 462 transcripts displayed a greater abundance in immature females and of these, 188 (41%) were assigned putative functions based on their sequence similarity with *D. melanogaster* genes and their associated ontologies. A total of 349 transcripts were more abundant in mature females, of which 137 (39%) were assigned putative functions. Of these transcripts those that are related to reproduction, behaviour, chemoreception and immune processes are listed in Table S2 and Figure 1A illustrates their frequencies also in relation to broader functional categories such as metabolic processes.

Statistically significant (p<0.05 after Yekutieli false discovery rate correction) enriched biological process GO terms in the immature females included processes involved in development, regulation of growth, morphogenesis, nervous system development and activation, response to stimuli, movement, oogenesis and immune effector processes. Significantly enriched terms in the mature female included lipid, sugar and amino acid metabolic processes, the biosynthesis and regulation of hormones and pheromones, and sensory perception of chemical stimuli (Table S3).

### Transcriptional changes during male maturation

A total of 301 transcripts displayed differential abundance between immature and mature virgin males. Of the 119 transcripts that were more abundant in the immature males, 59 (50%) were assigned putative functions and of the 182 transcripts that were enriched in mature males, 124 (68%) were similarly annotated (Table S4, Figure 1B). In immature males, statistically significant enriched GO terms included metabolism of carbohydrates and proteins, protein transport, immune effector response, muscle contraction and flight behaviour (Table S5). Enriched terms in mature males were dominated by a variety of metabolic and biosynthetic processes including pheromone and hormone biosynthesis and regulation, and immune response processes.

### Female and male mating responsive genes

In the female head, 24 hours after mating, only 32 transcripts were found to change their abundance compared to mature virgin female heads of the same age; 21 were enriched and 11 (34%) were less abundant in the mated female head compared to the mature virgin female head (Figure 1C). Of the transcripts that were enriched in mated females and had assigned putative functions, one was related to chemoreception (HS3757, a homologue of *Obp19d* enriched 1.76 fold), and one to reproduction (FS1820, a homologue of *Ptp61F* enriched 2.11 fold). The enriched biological process GO terms in the mated females included regulatory processes related to sensory perception of chemical stimuli, amino acid and protein metabolism and transport (Table S6). Only two transcripts with lower abundance in mated female heads were assigned putative functions, one related to lipid metabolism and the other to development. No significantly enriched biological process GO terms were present in the unmated females.

In males, by contrast, mating induces a massive wave of transcriptional changes. A total of 264 transcripts displayed changes in abundance, 79 enriched and 185 with lower abundance in the mated male, compared to the virgin male. Thirty-eight (48%) of the enriched and only 39 (21%) of the lower abundance transcripts were assigned putative functions (Figure 1D, Table S7). Enriched GO terms in the mated males were dominated by numerous regulatory processes, axonogenesis and metabolic processes (Table S8). Significantly enriched GO terms in the virgin males belonged to only two metabolic processes: chitin catabolic process and melanin metabolic process.

### Validation of transcriptome data derived from microarray by real-time quantitative PCR

As a confirmation of the validity of the microarray results, a highly significant positive correlation (r = 0.816, p<0.001) was found between the microarray and qRT-PCR datasets derived from the abundance of twelve transcripts in immature, mature and mated males and females (Figure S1, Table S9).

### The effect of maturation and mating on immune response genes

In the microarray analyses no evident mating-induced activation of immune-response related genes was observed. This apparent lack of an immune response after mating in the medfly might, however, be the result of the expression of these genes being limited to the reproductive tract or fat body, rather than in the head. To test this hypothesis, qRT-PCR analyses were performed on heads and abdomens of immature, mature and mated individuals of both sexes in order to determine whether genes related to the immune response show changes in transcriptional activity in relation to sexual maturation and mating status.

**Figure 2:**
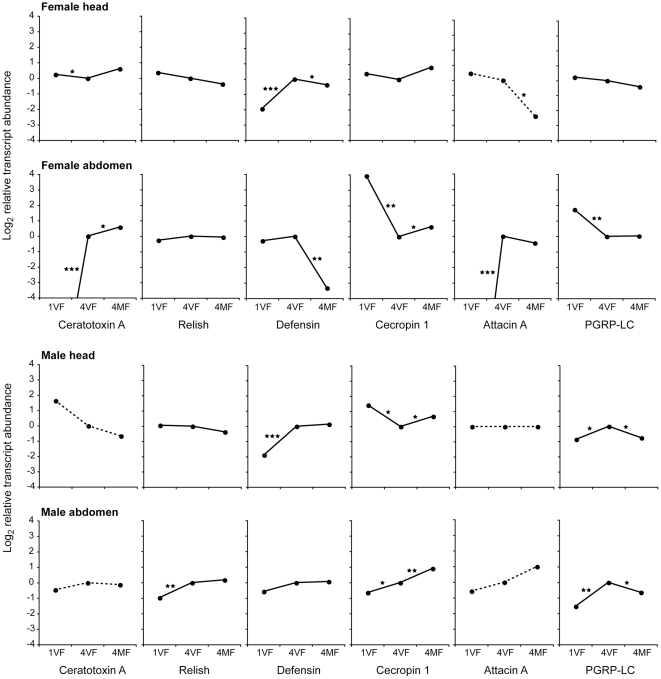
Differential transcript levels (Log_2_ transformed fold changes) of six immune genes in sexually immature, mature virgin, and mated medfly, compared to mature virgin medfly transcript levels. Transcript abundances were determined in immature (1V), mature virgin (4V) and mated (4M) female (upper) and male (lower) medfly heads and abdomens. Transcript abundances are expressed as ratios are compared to mature virgin levels (4V). Broken lines indicate that transcript abundances were very low. Stars indicate significant difference in transcript abundances (*P<0.05, **P<0.01, ***P<0.001, two-tailed *t*-test on three replicates) in the pairwise comparison between immature and mature virgin flies, or mature virgin and mated flies. Lines between data points are purely for ease of graphical interpretation and do not imply a continuous relationship between data points.

During maturation, the heads of mature virgin individuals of both sexes exhibited significantly higher (4-fold) levels of the *Defensin* gene transcript with respect to their immature counterparts, while *Ceratotoxin A* and *Attacin A* transcripts showed strong increases (several thousand fold) in the mature virgin female abdomen with respect to the immature females (Figure 2). Apart from the female abdomen, transcripts of *Attacin A* were detectable at very low levels (threshold cycle, C_T_≥34) in immature, mature and mated individuals of both sexes. Transcripts of *Cecropin 1* and *PGRP-LC* showed 15- and 3-fold lower abundance, respectively, on maturation in female abdomens, whereas in the male abdomen the abundance of these gene transcripts increased 1.6-fold and 2.6-fold, respectively, during maturation. A modest 2-fold increase in the transcript abundance of *Relish* was evident only in the mature virgin male abdomen with respect to the immature male. Thus, during maturation, massive changes in the transcriptional activity of immune-response-related genes were evident predominantly in the female abdomen (Figure 2).

Upon mating, only *Ceratotoxin A* and *Cecropin 1* showed significant, but very modest increases in transcript abundance in the female abdomen (1.5-fold). The only other immune-related genes to show significant changes in transcript abundance after mating in the female were *Defensin* which was less abundant, particularly in the abdomen (10-fold), and *Attacin A* that was less abundant in the female head, compared to virgin females, although the extent was difficult to quantify due to the very low transcript abundance (Figure 2). In mated males there was a modest increase in the *Cecropin 1* transcript abundance in the head and abdomen (1.6- and 1.9-fold respectively) and a decrease in *PGRP-LC* abundance in both body compartments (1.6- and 1.7-fold) compared to virgin males. Therefore, after mating, apart from a large reduction in *Defensin* transcript abundance in the female abdomen, only modest changes of transcript abundance were evident for the other immune-related genes (Figure 2).

## Discussion

*Ceratitis capitata* is a polyphagous *r*-selected insect that has evolved an opportunistic life history strategy. Each sex has different behavioural and physiological attributes that maximize their reproductive potentials. In order to analyse the molecular basis of these attributes, we used a microarray-based gene expression approach to determine the effects of maturation and mating on transcript abundance in each sex. Attention was focused largely on differentially represented transcripts related to reproduction, behaviour, sensory perception of chemical stimulus, and immune system processes. We chose these GO groups as, a priori, we expected changes in these system processes with maturation and mating. We are confident in the microarray-based transcriptome results as we obtained a highly significant positive correlation of the microarray dataset with qRT-PCR results for a subset of genes.

### Maturation induces radical changes in transcript abundance in female medfly

Medfly females need to mature for two to three days before they become sexually receptive [49]. In this period they tend to actively disperse in search of plant hosts for foraging and that will eventually act as oviposition sites. Gonad development is rapid, indeed vitellogenesis is thought to commence prior to eclosion of the female from the pupa [50]. During this short maturation period, the female needs to feed profusely on carbohydrates and proteins in the form of free amino acids, for her own development and for that of her eggs [51]. This behavioural physiology correlates well with the transcriptional results, where in the immature females the significantly enriched GO biological process categories were related to growth and development, including that of the nervous and immune systems. Metabolic categories, and in particular, fatty acid metabolism, were enriched, a reflection of the high energy requirements for processes essential for female reproductive success, such as foraging, nutrient storage and egg development [51]. The homologues of *Darkener of apricot*, *singed*, *zipper* and *bunched*, all involved in oocyte development and maturation [52]–[55], were enriched in the immature females compared to mature females.

Medfly foraging behaviour involves memory/learning, visual and olfactory functions [51]. This correlates with the transcriptional enrichment of homologues of the genes *crammer*, *scribbler* and *staufen*, involved in synaptic growth, synapse-specific modification and long-term memory formation in *D. melanogaster* [56], [57]. There was enrichment of transcripts of the *giant fibre A* gene which encodes a nicotinic acetylcholine receptor that mediates fast signalling at synapses in the central nervous system. This gene is expressed in the giant fibre that connects the visual nervous system in the head to the leg and flight neural circuits in the thorax, integrates visual input and activates the leg and flight motor neurons sequentially, resulting in a rapid escape jump [58]. The development of this escape response may be of particular importance in female medfly as it may enable them to escape predators that await them on fruit hosts [59], or perhaps it may help them avoid unsolicited mating attempts. The *smi35A* gene, also enriched, is thought to code for a dual specificity tyrosine-phosphorylation-regulated kinase involved in brain development and smell-recognition in particular [60].

With regard to chemoreception, transcripts of two odorant binding protein (Obp) gene homologues were enriched, *Obp8a* and *Obp19d* (HS3757). In *D. melanogaster*, *Obp8a* has been shown to be involved in the response to the aliphatic fruit odorant, hexanol [61], whereas *Obp19d* is associated with nutrient sensing, synaptic transmission and resistance to starvation stress [61]. Thus, we can hypothesize that in the medfly, these two *Obp* genes may be related to the localisation of food resources during foraging activity. Apart from these chemosensory-related transcripts, three *male-specific serum polypeptide* (*MSSP*) transcripts were enriched up to almost nine-fold. These *MSSP* genes were initially thought to be expressed only in the fat body of adult males, however, subsequently low levels of transcripts and their products were identified in the fat body of females and in the midgut of both sexes. They belong to the Minus-C subfamily of odorant binding proteins and are thought to be involved in the transport of volatile substances or other hydrophobic molecules [62], [63].

A number of genes related to immunity displayed enriched transcript abundance in immature females compared to mature females. Among these is the Imd pathway transcription factor *Relish* that induces the synthesis of the antimicrobial peptides *Defensin*, *Cecropin*, *Diptericin*, *Attacin* and *Metchnikowin* in *Drosophila* [64], [65]. The higher abundance of transcripts involved in the immune response to bacterial, fungal and viral infections such as *Cecropin* 1, *thiolester containing protein IV* and *Dicer-2* [47], [66]–[68] may be related to the nature of the food sources of the medfly, which are often rich in bacterial and fungal communities. Indeed, the adults feed on sugars from overripe and rotting fruit, honeydew and nectar and obtain proteins from protein-rich fruit, bird faeces and bacterial films on leaf surfaces and decomposing fruit [6].

Upon reaching sexual maturity, and having located appropriate plant hosts, the female's movements become less dispersive and she becomes receptive to courting males. In the meantime, foraging continues to be a major activity to sustain the energetic requirements of continuous egg development and oviposition [51]. In mature females, changes in transcript abundance is seen to underlie this behaviour as it is biased towards intense metabolic and biosynthetic activity. This biosynthetic activity may also involve the synthesis of cuticular sex pheromones which may represent important stimuli during courtship. In turn, transcripts related to the sensory perception of chemical stimulus GO category were also enriched, and this is likely to be associated with the female's requirement to detect pheromone-emitting males within the leks.

As in the immature females, many of the enriched transcripts in mature females are implicated in oogenesis and include *armadillo*, *spindle-B* and *Cdc42*. Of these, *Cdc42* is involved in the regulation of cell shape both in follicle cell development, the nervous system and the eye [69]. Two vitellogenin (*Vg2*) precursor genes show relatively large increases in transcript abundance in mature females. These genes are expressed by the fat body, secreted into the haemolymph and subsequently sequestered by oocytes [70]. Vitellogenins facilitate the transport of carbohydrates, lipids and other nutrients to the ovaries. Given that the head contains fat body, it was not unusual that *Vg* transcripts were detected therein. Indeed, expression of vitellogenin genes and their *yolk protein* homologues have been described in the *An. gambiae* and *D. melanogaster* head respectively [17], [71], [72].

Homologues of the *desat1* gene showed enriched transcript abundance in mature females. In *Drosophila desat1* is expressed in the fat body and is involved in the biosynthesis of unsaturated hydrocarbons on the cuticle of mature flies of both sexes [73]. These hydrocarbons represent sex pheromones and are required for mate discrimination by males in the absence of visual and other non-chemical cues. Also enriched was a homologue of *takeout* (*to*). The *to* gene possesses an insect juvenile hormone (JH) binding domain [74] and may act by modulating the circulating JH level [75]. In many insects, after mating the level of JH increases, stimulating egg development [76], [77]. However, given that mature virgin medfly females develop mature eggs and even oviposit [23], the stimulation of egg development by changes in JH levels, as a result of *to* enrichment, may be independent of any requirement for mating. In addition, *to* regulates feeding behaviour in a circadian sensitive manner [78]–[82].

The enrichment of six odorant binding protein transcripts, *Obp19a*, *Obp19b*, a second homologue of *Obp19d* (HC2265), *Obp56h* and two homologues of *Obp83a* (HS1065 and HC2536), may be related to host location, feeding and detection of the male pheromone. In *Drosophila*, *Obp19a* and *Obp19b* are involved in the response to aromatic and aliphatic odorants present in fruits [61], [83], [84], while *Obp56h* may have both olfactory and gustatory roles as it is expressed in the antenna and the pharyngeal organs [83], [85]. The enrichment of transcripts of two *Obp83a* homologues (HS1065 and HC2536), which incidentally also display enrichment in mature virgin males (compared to immature males), may be related to the perception of pheromones emitted by courting males. In *Drosophila*, *Obp83a* is expressed in the antenna and exhibits sexual dimorphism in its response to odorants [85]. *Pinocchio* (*Pino*) is another chemosensory-related gene, which contributes to early chemical recognition processes in the olfactory and immune system and may mediate the removal of xenobiotics such as odorants and bacterial toxins [86], [87]. Moreover, *Pino* is thought to negatively regulate the expression of *Obp8a*, as *Pino*-deficient *Drosophila* have elevated *Obp8a* expression [86]. This regulatory function may also exist in the medfly, as mature females with enrichment of *Pino* transcripts had lower *Obp8a* transcript abundances.

Mature females also showed higher transcript levels of immune-response related genes active against bacterial and fungal infections, *Defensin* [88] and *thiolester containing protein II* (*TepII*) [67], than immature females.

### Maturation induces modest changes in transcript abundance in male medfly

During their brief maturation period, before they become sexually active [89], male medfly spend a large proportion of their time feeding to accumulate energy and nutrient reserves upon on which their reproductive success will depend [6], [51]. Mirroring this, at the transcriptome level, immature males show significant enrichment of GO terms involved in various regulatory activities, protein and carbohydrate metabolism, energy production, muscle activity and flight behaviour. The enrichment of terms related to muscle contraction and flight behaviour is interesting given the importance of complex wing vibration movements in pheromone emission and during close range courtship behaviour in medfly [4]. In this regard, homologues of the *α-actinin*, and the *tropomyosin 1* genes, which are expressed principally in the indirect flight muscles and are essential for muscle contraction [90], [91], were enriched about two-fold in immature males. Homologues of *Obp8a* and *Obp19d* (HS3757), showed changes in transcript abundances that mirrored those found in immature females (compared to mature females), again suggesting their involvement in the location of food resources. Similarly, *smi35A* and *crammer*, both involved in the development of the nervous system, memory formation and smell-recognition, showed similar changes in transcript abundance as in their female counterparts.

As mature males are actively involved in lek formation, which involves the emission of pheromones and the courting of females, it is of no surprise that they show significant enrichment of GO terms involved in numerous metabolic and biosynthetic processes including pheromone synthesis. These processes are an indication of the high energetic investment required for flight to the lek site, courtship, and pheromone production. The enrichment of terms related to eye pigmentation is also interesting, given that the sexually dimorphic bright colouring of the male's eyes are thought to send visual signals to the female during courtship behaviour [4].

Significantly higher transcript abundances of *vitellogenin* genes *1* and *2* were observed in mature males compared to their immature counterparts. This differs from the expression of Yolk proteins 1 and 2 in *D. melanogaster*, which are limited almost exclusively to females [72]. However, the expression of these genes should not be considered female-specific as they have been reported in males of several insect species including honeybees, cockroaches and moths [70], [92], [93]. Vitellogenins have also been implicated with the transport of various molecules including sugars, lipids and hormones in insects [94].

As in their female counterparts, mature males displayed enrichment of two transcripts with significant sequence similarity to *Obp83a* (HC2536 and HS1065). Changes in the abundances of these transcripts may be correlated to the onset of participation in leks and active courtship behaviour. As the sex pheromone emitted by males attracts not only females, but also other males to participate in the lek [4], the similar changes in transcript abundance of these genes in both sexes is not entirely unexpected.

Transcripts related to the innate humoral response system tended to increase in abundance with male maturation. As in the mature females, the transcripts of *Defensin*, *CG16799*, *Spn27A*, *TepII* and *modSP* were more abundant in mature male individuals compared to their immature counterparts.

### Female mating does not trigger major changes in transcript abundances

Upon mating, medfly females tend to become unreceptive to courting males, although a proportion of females do remate [25]. The mated female continues to feed profusely as she needs to secure the energy and nutrients required for continued egg development and oviposition. Surprisingly, the only enriched transcripts in the mated female heads were *protein tyrosine phosphatase 61F* (*ptp61F*) and a homologue of *Obp19d* (HS3757). The altered abundances of these transcripts may be the result of the action of male accessory gland proteins, and/or sperm [29], [33]. The enrichment of *ptp61F* transcripts may be related to behavioural changes in the response of mated females to courting males as, in *Drosophila*, the protein associates with *dreadlocks* an adaptor protein involved in axon guidance, GPCR protein signalling pathway and the insulin receptor signalling pathway [95].

Unlike the medfly, *Drosophila* females down-regulate expression of *Obp19d* after mating [11]. Perhaps the modulation of *Obp19d* in the medfly may contribute to post-mating changes in the females' attraction or receptivity to courting males [96]. *Obp19d* may also be implicated in the detection of the host-marking pheromone deposited on the fruit by other females after oviposition, that acts as a deterrent to further oviposition [97]. However, given that the same *Obp19d* homologue (HS3757) was also enriched in immature medfly individuals of both sexes and also in mated males (compared to virgin males) it is more likely, as previously suggested, that this gene is related to foraging behaviour. No immune-related genes showed changes in transcript abundance between mature virgin and mated females.

### Mating induces considerable changes in transcript abundance in male medfly

Male medfly can potentially continue to court and mate throughout their life. Courtship involves a complex series of activities such as lek participation, male-male conflict, pheromone emission and complex wing movements, all of which may eventually lead to mating that can last several hours. After this intense energy expenditure, the male needs to replenish his depleted reserves by foraging [98]. Again this behaviour is mirrored by the enricment of GO terms related to numerous biosynthetic and regulatory processes and nervous system development. As in females, mated males showed enrichment of *Ptp61F* transcripts responsible for changes in the synaptic organisation in the nervous system related to learning, perhaps as a result of stimuli received during the courtship process [99]. Thus, after an initial mating, males may perceive and process stimuli from courting females more rapidly and may subsequently become more successful in gaining matings in situations where competition for females is intense [19]. In this context, a homologue of *derailed*, involved in synaptic growth and synapse-specific modification, was also enriched [100]. The homologue of *double-time* influences the oscillations of the clock genes *period* and *timeless* [101], [102], and may be implicated in time-dependent activities, such as mating and feeding in the medfly [103].

Male medfly display a variety of aggressive behaviours, related to competition for access to females and to the defence of favourable positions in the lek [4]. The gene *Basigin* (*Bsg*), which in *Drosophila* is principally expressed in the fat body, has been implicated in inter-male aggressive behaviour [104]. As knockout of *Basigin* in *Drosophila* is associated with increased inter-male aggression [104], one might expect that the lower abundance of its transcripts in mated male medfly might be correlated to increased aggressiveness, which may result in higher sexual competitiveness.

Given the importance of visual cues in medfly courtship behaviour, it was interesting that transcripts of the *CDP diglyceride synthetase* (*CdsA*) gene were less abundant in mated males. This gene is under the influence of the photocycle and is thought to promote daily variations in visual sensitivity [105]. Perhaps, in the time period during which courtship occurs, the acuity of the male eyes is optimized in order to detect subtle cues in female behaviour.

### Maturation, mating status and immune response in male and female medfly heads and abdomens

In our transcriptome analyses, although changes in transcript abundance of immune-response related genes were observed during the maturation of both sexes, no evident mating-induced activation effect was observed. This is in contrast to previous studies on *D. melanogaster* [10], [28], [106]. In *Drosophila* the immune response is activated in mated females by the sperm and seminal fluid components. It has been suggested that this immune response is part of a sexually antagonistic arms race in which the male produces increasingly potent signal molecules that modify the behaviour and physiology of the female away from reproductive receptivity towards fecundity. For the females these signalling molecules may induce suboptimal reproductive behaviour as they may favour short-term fecundity over long-term reproductive investment. Thus, the female immune system may have been co-opted to sequester the male signalling molecules to limit their effects [106]–[108].

The apparent lack of an immune response after mating in the medfly might, however, be the result of the expression of these genes being limited to the reproductive tract or fat body, rather than in the head. To test this hypothesis, qRT-PCR assays were performed on six genes involved in the immune response on heads and abdomens of immature, mature virgin and mated males and females (Figure 2). Although highly significant changes in transcript abundance of several of these genes were evident between immature and mature virgin individuals upon mating, only *Ceratotoxin A* and *Cecropin 1* showed significant but very modest enrichment in the female abdomen. This *Ceratotoxin A* expression profile is congruent with that described in a previous study in which it was shown to be expressed in the accessory glands of sexually mature medfly females and to be enhanced by mating [48]. As *Ceratotoxin A* is also present on the surface of laid eggs it has been suggested that it may function in protecting early larval development from bacterial infection. The only other immune-related genes to show changes after mating in the female were *Defensin* and *Attacin A* which displayed lower transcript abundances, particularly in the abdomen and head, respectively. In males there was only a modest enrichment of *Cecropin 1* and reduced abundance of *PGRP-LC* transcripts in both body compartments after mating.

This, albeit limited survey, suggests that mating in medfly does not appear to activate immune gene expression. In this sense the medfly displays a greater similarity to its more distant evolutionary relatives, *A. gambiae* and *A. mellifera*, which also lack this post-mating immune-response, than to *D. melanogaster* [15], [17].

### Conclusions

A substantial number of transcripts displayed significant changes in abundance during maturation in both sexes and these changes generally mirrored the physiological and behavioural changes that accompany maturation in male and female medfly. Despite extensive post-mating transcriptional changes in the male, changes in the female were surprisingly modest. The current study was restricted to the head transcriptome which may be a limitation, but the observation that unmated medfly females are just as prolific egg-layers as their mated counterparts [23] does suggest that virgin females are literally ‘poised’ for egg fertilization. Virgin medfly females not only develop mature eggs but they also initiate oviposition behaviour [23]. This is quite different to *D. melanogaster*, where the transfer of accessory gland proteins (Acps) to females during copulation is essential for egg development, egg maturation and the induction and maintenance of egg laying [14], [109], [110]. This suggests that, unlike *Drosophila*, *C. capitata* male accessory gland proteins transferred during copulation have little or no effect on egg production. The medfly is an *r*-strategist and the selective benefits to a virgin female having mature eggs ready for fertilisation and oviposition immediately upon mating may counter-balance the costs of egg production in the unlikely event that the female remains unmated [23]. Male seminal proteins do, however, appear to be important in modifying the receptivity of female *C. capitata* to remating [27]. Behavioural assays have shown that mating, or the injection of male accessory gland extracts into virgin females, results in a change in the olfactory behaviour of the female from a preference for male pheromone to host fruit odours [30]. The lack of apparent mating-induced immune responses in the medfly may be a reflection of the minor influence of male signalling proteins on female reproductive physiology, and the consequent lack of the sexually antagonistic arms race that is present in *Drosophila* [106]–[108].

Knowledge of the intricacies of the processes related to maturation and mating provides powerful targets for manipulating the reproduction of these insects in wild populations. As the medfly represents a model species for tephritid fruit flies, which include numerous important invasive agricultural pests throughout the world, the potential benefit of this knowledge will not be restricted to the medfly alone. In addition to increasing our understanding of the molecular machinery behind these biological processes, our findings may provide insights into the evolutionary processes that have led to the invasive success of this tephritid species.

## Supporting Information

Figure S1 Validation of microarray-assayed changes in transcript abundance with quantitative real-time RT-PCR for 12 medfly genes. The mean values for the transcript abundance data (log_2_ ratio) for 12 genes from the four assays (Table S9) obtained by microarray analysis are plotted against the corresponding mean expression values obtained with real-time RT-PCR from three biological replicates of each experiment. The Pearson correlation coefficient, r = 0.816 which is highly significant at p = 2.8e-12, and the slope of the regression line (m = 1.18) demonstrate a high degree of correlation between the two assays.
(TIF)

Table S1 Primers used for qRT-PCR analyses.
(DOC)

Table S2 Transcripts that change in abundance in mature virgin females compared to immature virgin females.
(DOC)

Table S3 Significantly enriched biological process gene ontology annotations among transcripts that showed changes in abundance in mature virgin female heads compared to immature female heads.
(DOC)

Table S4 Transcripts that change in abundance in mature virgin males compared to immature virgin males.
(DOC)

Table S5 Significantly enriched biological process gene ontology annotations among transcripts that showed changes in abundance in mature virgin male heads compared to immature male heads.
(DOC)

Table S6 Significantly enriched biological process gene ontology annotations among transcripts that showed changes in abundance in mated female heads compared to mature virgin female heads.
(DOC)

Table S7 Transcripts that change in abundance in mated males with respect to mature virgin males.
(DOC)

Table S8 Significantly enriched biological process gene ontology annotations among transcripts that showed changes in abundance in mated male heads compared to mature virgin male heads.
(DOC)

Table S9 Correlation of microarray changes of transcript abundance with real-time qRT-PCR for 12 genes (log_2_ transformed fold expression).
(DOC)
