# Supplementary figures and images for: Transcriptome Profiling of Sexual Maturation and Mating in the Mediterranean Fruit Fly, Ceratitis capitata
Source: PLoS One. 2012 Jan 27;7(1):e30857. doi: 10.1371/journal.pone.0030857 (PMC3267753; doi:10.1371/journal.pone.0030857)

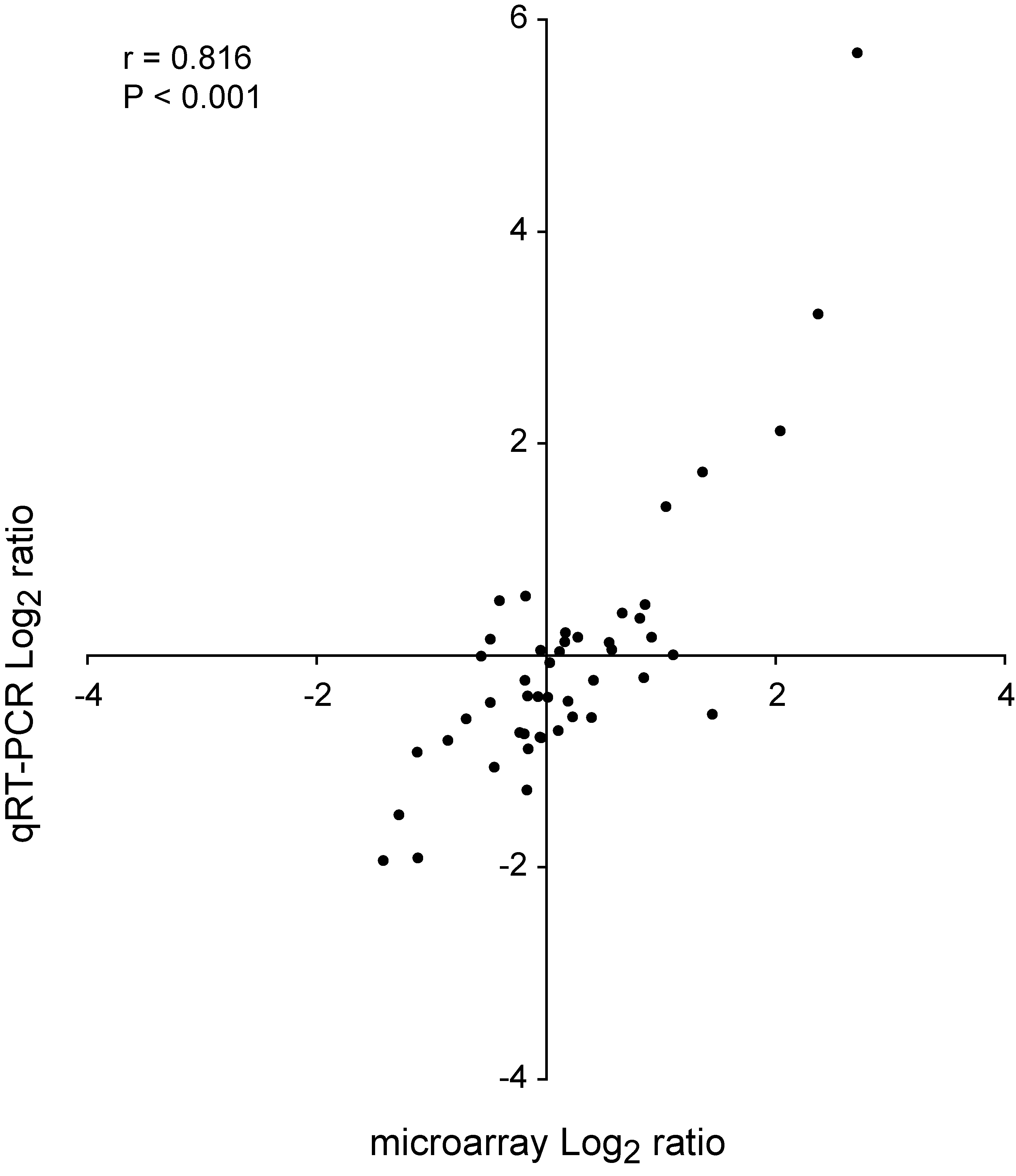

Supplement: Figure S1 — Validation of microarray-assayed changes in transcript abundance with quantitative real-time RT-PCR for 12 medfly genes. The mean values for the transcript abundance data (log2 ratio) for 12 genes from the four assays (Table S9) obtained by microarray analysis are plotted against the corresponding mean expression values obtained with real-time RT-PCR from three biological replicates of each experiment. The Pearson correlation coefficient, r = 0.816 which is highly significant at p = 2.8e-12, and the slope of the regression line (m = 1.18) demonstrate a high degree of correlation between the two assays. (TIF) [file pone.0030857.s001.tif]
