# Supplementary material for: Transcriptome Profiling of Sexual Maturation and Mating in the Mediterranean Fruit Fly, Ceratitis capitata
Source: PLoS One. 2012 Jan 27;7(1):e30857. doi: 10.1371/journal.pone.0030857 (PMC3267753; doi:10.1371/journal.pone.0030857)
Supplement: Table S1 — Primers used for qRT-PCR analyses. (DOC) [file pone.0030857.s002.doc]

Supplementary Table 1: Primers used for qRT-PCR analyses

| **Sequence ID (gene)** | **forward primer** | **reverse primer** |
| --- | --- | --- |
| FS831 (*GAPDH2*) | ggtcgcatcggtcgtctgg | gctgaaacggtgcccttgaaac |
| S67872 (*G6PDH*) | cggacgagcaggcaaaatatg | agacggacggcggtaagg |
| FC2009 (*RpL13A*) | gctgtacgaggcatgattcc | cagacacgacgacgcttg |
| HC321 (*takeout*) | taaagcaagaggattcggcaaag | cccacccattgaagtatcatatcg |
| FS1844 (*smi35A*) | gcagccattacaacgacaacc | ccaaatcggagacagatgaagtg |
| HC2068 (*MSSPγ1*) | gttttcaccaaatggggagttttc | gccgattatttcgtcaatctcttc |
| HC1570 (*Obp28a*) | tgccgctgactccgacattc | tccgcatcgccgtctgtatatg |
| HC1629 (*Obp69a*) | tgtattaaagaaactggcgtcacc | ttctacaatcggcacaaattcagg |
| HC2265 (*Obp19d*) | tgatgaatgacgatggcaaaatgg | atcttcagcagcctcgcaatg |
| HS3757 (*Obp19d*) | gcttgtctgcggcttaatcatc | gcatttacctttgtcagtgtttgg |
| HC2536 (*Obp83a*) | gagaaaactggcgtaagtgaagag | caccattatcatccaccacatcg |
| HC1181 (*Defensin*) | ttggaatcctctgtgctttatgc | cgctgtgatttacgccgaag |
| FC1457 (*Relish*) | acaaagttctcaatgcccacaatg | gttccttaacagcgatatgtagtgc |
| FS1820 (*Ptp61F*) | tattacaatttcactacacaac | gacagcaatcaactaagc |
| AJ272446 (*Ceratotoxin A*) | gtggttaaacggagtattggtagc | aacgggtagagcagcctttg |
| X70030 (*Cecropin 1*) | gcgggttggctgaagaag | cggtggctgcgacattag |
| FC614 (*Attacin A*) | aaagtgtctacctctcgtttctgg | gcatagtagccactcaagtatcgc |
| HC731 (*PGRP-LC*) | gcacacaccaaaggctacaatc | cacccaaacgaagaccctcatc |

Sequence ID refers to assembled EST sequence or GenBank accession numbers for G6PDH, *Ceratotoxin A* and *Cecropin 1*.
