# Supplementary material for: Transcriptome Profiling of Sexual Maturation and Mating in the Mediterranean Fruit Fly, Ceratitis capitata
Source: PLoS One. 2012 Jan 27;7(1):e30857. doi: 10.1371/journal.pone.0030857 (PMC3267753; doi:10.1371/journal.pone.0030857)
Supplement: Table S2 — Transcripts that change in abundance in mature virgin females compared to immature virgin females. (DOC) [file pone.0030857.s003.doc]

Supplementary Table 2**:** Transcripts that change in abundance in mature virgin females compared to immature virgin females. Up and down arrows refer to an increase or a decrease in transcript abundance in the mature females compared to immature females. Unless otherwise stated BLASTX hits and the associated e-values refer to *Drosophila melanogaster* sequences

| **GO category** | **Transcript** | **fold-change** | **Best BLASTX hit** | ***e*-Value** |
| --- | --- | --- | --- | --- |
| Reproduction | HS3621 | 2.77 ↓ | *zipper* (*zip*) | 7e-75 |
|  | HC1646 | 1.85 ↓ | *growl* | 6e-26 |
|  | HC1678 | 1.75 ↓ | *Ran-binding protein M* (*RanBPM*) | 8e-81 |
|  | HS1366 | 2.59 ↓ | *Ran-binding protein M* (*RanBPM*) | 8e-41 |
|  | FC1803 | 1.91 ↓ | *polyA-binding protein* (*pAbp*) | 1e-127 |
|  | FC57 | 2.37 ↓ | *Gef26* | 1e-17 |
|  | HS2667 | 2.25 ↓ | *kismet* (*kis*) | 2e-44 |
|  | FS711 | 2.47 ↓ | *singed* (*sn*) | 1e-141 |
|  | HS368 | 3.17 ↓ | *Darkener of apricot* (*Doa*) | 1e-12 |
|  | FS2657 | 3.24 ↓ | *Darkener of apricot* (*Doa*) | 5e-30 |
|  | FC1773 | 2.54 ↓ | *Topoisomerase 1* (*Top1*) | 1e-59 |
|  | HC943 | 2.95 ↓ | *Protein kinase 61C* (*Pk61C*) | 7e-42 |
|  | FC1682 | 2.30 ↓ | *Protein kinase 61C* (*Pk61C*) | 8e-75 |
|  | HC2573 | 1.79 ↓ | *Tropomyosin 1* (*Tm1*) | 1e-131 |
|  | FC1840 | 1.96 ↓ | *Ribonuclear protein at 97D* (*Rb97D*) | 5e-71 |
|  | HS2341 | 1.84 ↓ | *Clathrin heavy chain* (*Chc*) | 8e-83 |
|  | FS960 | 2.10 ↓ | *four wheel drive* (*fwd*) | 1e-125 |
|  | HC1773 | 2.63 ↓ | *capicua* (*cic*) | 5e-66 |
|  | FS1820 | 1.84 ↓ | *Protein tyrosine phosphatase 61F* (*Ptp61F*) | 8e-69 |
|  | FS1027 | 2.49 ↓ | *transforming acidic coiled-coil protein* (*tacc*) | 2e-17 |
|  | FS1163 | 1.87 ↓ | *Chromodomain-helicase-DNA-binding protein 1* (*Chd*) | 1e-57 |
|  | HC1385 | 2.03 ↓ | *bunched* (*bun*) | 8e-35 |
|  | HC1112 | 3.85 ↑ | *Bactrocera dorsalis* vitellogenin 2 precursor | 1e-145 |
|  | HC939 | 3.67 ↑ | *Bactrocera dorsalis* vitellogenin 2 precursor | 1e-133 |
|  | HC1106 | 2.26 ↑ | *armadillo* (*arm*) | 3e-24 |
|  | HS3173 | 2.15 ↑ | *easter* (*ea*) | 1e-36 |
|  | FS48 | 1.96 ↑ | *spindle B* (*spn-B*) | 3e-23 |
|  | HC2691 | 2.31 ↑ | *Cdc42* | 1e-107 |
|  | FS927 | 1.90 ↑ | *desat1* | 3e-29 |
|  | FC2022 | 2.07 ↑ | *desat1* | 0 |
|  | HS1044 | 1.74 ↑ | *desat1* | 1e-125 |
|  | HC2468 | 2.02 ↑ | *desat1* | 1e-123 |
| Behaviour | HC753 | 2.27 ↓ | *crammer* (*cer*) | 7e-27 |
|  | FC988 | 2.07 ↓ | *α-actinin* (*Actn*) | 1e-101 |
|  | HC1891 | 1.85 ↓ | *α-actinin* (*Actn*) | 2e-51 |
|  | HC2597 | 1.98 ↓ | *α-actinin* (*Actn*) | 0 |
|  | FS2268 | 1.90 ↓ | CG42684 | 3e-16 |
|  | HS3303 | 2.42 ↓ | *adenosine deaminase acting on RNA* (*Adar*) | 1e-103 |
|  | HC1867 | 2.00 ↓ | *giant fibre A* (*gfA*) | 4e-51 |
|  | HC1989 | 1.90 ↓ | *Gp150* | 3e-10 |
|  | HC837 | 1.83 ↓ | *Gp150* | 2e-10 |
|  | FS2586 | 1.74 ↓ | *Gp150* | 2e-10 |
|  | FS1844 | 4.84 ↓ | *smell impaired 35A* (*smi35A*) | 1e-26 |
|  | HS581 | 2.52 ↓ | *smell impaired 35A* (*smi35A*) | 3e-25 |
|  | FS1375 | 1.75 ↓ | *scribbler* (*sbb*) | 2e-12 |
|  | FS1467 | 1.74 ↓ | *Dopamine N acetyltransferase* (*Dat*) | 5e-81 |
|  | FS2959 | 1.93 ↓ | *no ocelli* (*noc*) | 1e-08 |
|  | HC2000 | 2.04 ↓ | *staufen* (*stau*) | 8e-51 |
|  | FS1691 | 1.81 ↓ | CG9171 | 1e-129 |
|  | FS1695 | 2.19 ↑ | *Pinocchio* (*Pino*) | 2e-17 |
|  | HS2483 | 2.03 ↑ | *takeout* (*to*) | 2e-32 |
|  | HS3861 | 1.81 ↑ | CG5946 | 1e-101 |
| Chemoreception | HC2068 | 5.31 ↓ | *C. capitata male specific serum polypeptide γ1* (*MSSPγ1*) | 2e-63 |
|  | HC2316 | 8.89 ↓ | *C. capitata male specific serum polypeptide γ1* (*MSSPγ1*) | 8e-62 |
|  | HC984 | 6.12 ↓ | *C. capitata male specific serum polypeptide γ1* (*MSSPγ1*) | 3e-61 |
|  | FS806 | 2.15 ↓ | *Odorant-binding protein 8a* (*Obp8a*) | 8e-15 |
|  | HS3757 | 2.01 ↓ | *Odorant-binding protein 19d* (*Obp19d*) | 4e-12 |
|  | HC2265 | 2.16 ↑ | *Odorant-binding protein 19d* (*Obp19d*) | 1e-26 |
|  | HC1947 | 1.78 ↑ | *Odorant-binding protein 19a* (*Obp19a*) | 1e-39 |
|  | HS1065 | 2.67 ↑ | *Odorant-binding protein 83a* (*Obp83a*) | 2e-59 |
|  | HC2536 | 2.19 ↑ | *Odorant-binding protein 83a* (*Obp83a*) | 4e-62 |
|  | HS1079 | 1.81 ↑ | *Odorant-binding protein 19b* (*Obp19b*) | 5e-26 |
|  | HC1321 | 2.35 ↑ | *Odorant-binding protein 56h* (*Obp56h*) | 1e-15 |
| Immune system | FC1457 | 1.75 ↓ | *Relish* (*Rel*) | 7e-51 |
|  | HS653 | 1.84 ↓ | *longitudinals lacking* (*lola*) | 3e-18 |
|  | HS3067 | 3.34 ↓ | *Dicer-2* (*Dcr-2*) | 4e-77 |
|  | FC713 | 1.81 ↓ | *Dicer-2* (*Dcr-2*) | 1e-106 |
|  | HC853 | 1.92 ↓ | *Thiolester containing protein IV* (*TepIV*) | 8e-59 |
|  | HC1093 | 1.76 ↓ | *virus induced RNA 1* (*vir-1*) | 2e-08 |
|  | HS1598 | 1.79 ↓ | *Mig-2-like* (*Mtl*) | 3e-82 |
|  | X70030.1 | 4.83 ↓ | *C. capitata Cecropin 1* (*CcCec1*) | 0 |
|  | HC1181 | 2.90 ↑ | *Defensin* (*Def*) | 7e-12 |
|  | HS2632 | 2.06 ↑ | *CG16799* | 6e-29 |
|  | HS3323 | 4.69 ↑ | *Thiolester containing protein II* (*TepII*) | 5e-27 |
|  | FS318 | 1.78 ↑ | *p38b* | 1e-141 |
|  | FS2005 | 1.89 ↑ | *modular serine protease* (*modSP*) | 2e-15 |
|  | HC2183 | 1.83 ↑ | *serpin-27A* (*Spn27A*) | 5e-24 |
