# Supplementary material for: Transcriptome Profiling of Sexual Maturation and Mating in the Mediterranean Fruit Fly, Ceratitis capitata
Source: PLoS One. 2012 Jan 27;7(1):e30857. doi: 10.1371/journal.pone.0030857 (PMC3267753; doi:10.1371/journal.pone.0030857)
Supplement: Table S3 — Significantly enriched biological process gene ontology annotations among transcripts that showed changes in abundance in mature virgin female heads compared to immature female heads. (DOC) [file pone.0030857.s004.doc]

**Supplementary Table 3: Significantly enriched biological process gene ontology annotations among transcripts that showed changes in abundance in mature virgin females compared to immature females**

| **Expression** | **Gene Ontology Term** | **Significant^1^** | **Annotated^2^** | **FDR-adjusted P-value** |
| --- | --- | --- | --- | --- |
| Enriched in immature females | multicellular organismal development | 60 | 1002 | 3.2e-02 |
|  | multicellular organismal process | 82 | 1317 | 9.6e-04 |
|  | developmental process | 70 | 1145 | 8.7e-03 |
|  | response to stimulus | 56 | 894 | 2.e-02 |
|  | system process | 23 | 274 | 2.5e-02 |
|  | axonogenesis | 15 | 147 | 2.7e-02 |
|  | axon guidance | 12 | 104 | 2.7e-02 |
|  | neuron recognition | 8 | 47 | 1.3e-02 |
|  | cell projection organization | 20 | 233 | 3.6e-02 |
|  | cellular component morphogenesis | 23 | 287 | 4.1e-02 |
|  | locomotion | 20 | 232 | 3.4e-02 |
|  | neuromuscular synaptic transmission | 4 | 14 | 2.1e-02 |
|  | regulation of growth | 10 | 73 | 1.9e-02 |
|  | negative regulation of microtubule polymerization or depolymerization | 5 | 19 | 1.2e-02 |
|  | actin filament bundle assembly | 4 | 7 | 4.2e-04 |
|  | sarcomere organization | 4 | 10 | 4.2e-03 |
|  | chitin-based embryonic cuticle biosynthetic process | 3 | 8 | 2.1e-02 |
|  | immune effector process | 5 | 14 | 1.7e-03 |
|  | defense response to virus | 3 | 7 | 1.4e-02 |
|  | negative regulation of Ras protein signal transduction | 3 | 8 | 2.1e-02 |
|  | muscle contraction | 4 | 17 | 4.5e-02 |
|  | triglyceride metabolic process | 3 | 9 | 3.2e-02 |
| Enriched in mature females | defense response | 9 | 83 | 1.3e-02 |
|  | cuticle hydrocarbon biosynthetic process | 4 | 5 | 3.6e-06 |
|  | cuticle development | 5 | 36 | 4.5e-02 |
|  | secondary metabolic process | 6 | 34 | 4.9e-03 |
|  | hormone metabolic process | 5 | 20 | 2.0e-03 |
|  | pheromone biosynthetic process | 4 | 6 | 1.9e-05 |
|  | lipid metabolic process | 14 | 153 | 4.5e-03 |
|  | fatty acid metabolic process | 6 | 40 | 1.3e-02 |
|  | fatty acid biosynthetic process | 5 | 21 | 2.7e-03 |
|  | carboxylic acid metabolic process | 15 | 209 | 3.5e-02 |
|  | carboxylic acid biosynthetic process | 8 | 57 | 4.0e-03 |
|  | sensory perception of chemical stimulus | 6 | 37 | 8.4e-03 |
|  | aromatic amino acid family metabolic process | 3 | 13 | 3.5e-02 |
|  | amine biosynthetic process | 6 | 49 | 4.2e-02 |
|  | spermidine metabolic process | 4 | 5 | 3.6e-06 |
|  | polyamine biosynthetic process | 3 | 5 | 4.7e-04 |
|  | aromatic amino acid family catabolic process | 2 | 5 | 2.1e-02 |

^1^Number of transcripts that show significantly increased abundance that are associated (directly or indirectly) with the Gene Ontology term

^2^Number of transcripts present on the microarray that are associated (directly or indirectly) with the Gene Ontology term
