# Supplementary material for: Transcriptome Profiling of Sexual Maturation and Mating in the Mediterranean Fruit Fly, Ceratitis capitata
Source: PLoS One. 2012 Jan 27;7(1):e30857. doi: 10.1371/journal.pone.0030857 (PMC3267753; doi:10.1371/journal.pone.0030857)
Supplement: Table S4 — Transcripts that change in abundance in mature virgin males compared to immature virgin males. (DOC) [file pone.0030857.s005.doc]

Supplementary Table 4: Transcripts that change in abundance in mature virgin males compared to immature virgin males. Up and down arrows refer to an increase or a decrease in transcript abundance in the mature males compared to immature males. Unless otherwise stated BLASTX hits and the associated e-values refer to *Drosophila melanogaster* sequences

| **GO category** | **Transcript** | **fold-change** | **Best BLASTX hit** | ***e*-Value** |
| --- | --- | --- | --- | --- |
| Reproduction | HS1366 | 1.86 ↓ | *Ran-binding protein M* (*RanBPM*) | 8e-41 |
|  | HS368 | 2.67 ↓ | *Darkener of apricot* (*Doa*) | 1e-12 |
|  | HC1112 | 4.31 ↑ | *Bactrocera dorsalis* vitellogenin 2 precursor | 1e-145 |
|  | HC770 | 8.26 ↑ | *Bactrocera dorsalis* vitellogenin 2 precursor | 7e-52 |
|  | HC1515 | 4.38 ↑ | *C. capitata* vitellogenin-1 precursor | 1e-136 |
|  | FS927 | 2.73 ↑ | *desat1* | 3e-29 |
|  | HC2468 | 2.23 ↑ | *desat1* | 1e-123 |
| Behaviour | FS1844 | 3.83 ↓ | *smell impaired 35A* (*smi35A*) | 1e-26 |
|  | HS581 | 2.09 ↓ | *smell impaired 35A* (*smi35A*) | 3e-25 |
|  | HC753 | 2.21 ↓ | *crammer* (*cer*) | 7e-27 |
| Chemoreception | HS3757 | 2.50 ↓ | *Odorant-binding protein 19d* (*Obp19d*) | 4e-12 |
|  | FS806 | 2.83 ↓ | *Odorant-binding protein 8a* (*Obp8a*) | 8e-15 |
|  | HS1065 | 1.94 ↑ | *Odorant-binding protein 83a* (*Obp83a*) | 2e-59 |
|  | HC2536 | 1.83 ↑ | *Odorant-binding protein 83a* (*Obp83a*) | 4e-62 |
| Immune system | HC1093 | 2.58 ↓ | virus induced RNA 1 (vir-1) | 2e-08 |
|  | HC1181 | 2.23 ↑ | *Defensin* (*Def*) | 7e-12 |
|  | HS3323 | 4.55 ↑ | Thiolester containing protein II (TepII) | 5e-27 |
|  | HS2632 | 1.87 ↑ | CG16799 | 6e-29 |
|  | HS3705 | 1.79 ↑ | *serpin-27A* (*Spn27A*) | 1e-56 |
|  | HC1288 | 1.91 ↑ | *modular serine protease* (*modSP*) | 4e-15 |
|  | FS2005 | 1.91 ↑ | *modular serine protease* (*modSP*) | 2e-15 |
