# Supplementary material for: Transcriptome Profiling of Sexual Maturation and Mating in the Mediterranean Fruit Fly, Ceratitis capitata
Source: PLoS One. 2012 Jan 27;7(1):e30857. doi: 10.1371/journal.pone.0030857 (PMC3267753; doi:10.1371/journal.pone.0030857)
Supplement: Table S5 — Significantly enriched biological process gene ontology annotations among transcripts that showed changes in abundance in mature virgin male heads compared to immature male heads. (DOC) [file pone.0030857.s006.doc]

Supplementary Table 5: Significantly enriched biological process gene ontology annotations among transcripts that showed changes in abundance in mature virgin males compared to immature males

| **Expression** | **Gene Ontology Term** | **Significant^1^** | **Annotated^2^** | **FDR-adjusted P-value** |
| --- | --- | --- | --- | --- |
| Enriched in immature males | larval central nervous system remodeling | 1 | 6 | 4.9e-02 |
|  | proteolysis | 13 | 308 | 2.6e-04 |
|  | aminoglycan metabolic process | 4 | 36 | 2.1e-03 |
|  | chitin metabolic process | 3 | 23 | 5.1e-03 |
|  | mitochondrial electron transport, succinate to ubiquinone | 1 | 6 | 4.9e-02 |
|  | negative regulation of Ras protein signal transduction | 2 | 8 | 3.7e-03 |
|  | immune effector process | 3 | 14 | 8.2e-04 |
|  | response to wounding | 2 | 13 | 1.5e-02 |
|  | chitin-based embryonic cuticle biosynthetic process | 2 | 8 | 3.7e-03 |
|  | actin filament bundle assembly | 3 | 7 | 5.3e-05 |
|  | cytoskeletal anchoring at plasma membrane | 3 | 17 | 1.6e-03 |
|  | flight behavior | 3 | 14 | 8.2e-04 |
|  | actin cytoskeleton reorganization | 3 | 18 | 2.0e-03 |
|  | regulation of exit from mitosis | 3 | 11 | 3.2e-04 |
|  | positive regulation of NFAT protein import into nucleus | 2 | 17 | 2.6e-02 |
|  | muscle contraction | 3 | 17 | 1.6e-03 |
|  | mesoderm development | 4 | 58 | 1.5e-02 |
|  | oligosaccharide biosynthetic process | 1 | 6 | 4.9e-02 |
|  | glycoside metabolic process | 1 | 5 | 3.6e-02 |
|  | regulation of lamellipodium assembly | 1 | 5 | 3.6e-02 |
| Enriched in mature males | catabolic process | 14 | 335 | 6.0e-03 |
|  | nucleotide metabolic process | 7 | 104 | 6.0e-03 |
|  | cellular nitrogen compound metabolic process | 22 | 774 | 3.0e-02 |
|  | cellular metabolic process | 45 | 2029 | 4.9e-02 |
|  | primary metabolic process | 48 | 2070 | 1.7e-02 |
|  | cellular catabolic process | 10 | 243 | 2.6e-02 |
|  | small molecule catabolic process | 11 | 87 | 5.8e-07 |
|  | nucleobase, nucleoside and nucleotide metabolic process | 8 | 115 | 2.4e-03 |
|  | lipid biosynthetic process | 5 | 63 | 9.7e-03 |
|  | biosynthetic process | 23 | 818 | 2.8e-02 |
|  | cellular lipid metabolic process | 12 | 113 | 1.3e-06 |
|  | plasma membrane organization | 2 | 16 | 3.2e-02 |
|  | cuticle hydrocarbon biosynthetic process | 2 | 5 | 1.3e-03 |
|  | regulation of hormone levels | 3 | 20 | 6.2e-03 |
|  | secondary metabolic process | 4 | 34 | 5.0e-03 |
|  | pheromone biosynthetic process | 2 | 6 | 2.5e-03 |
|  | fatty acid biosynthetic process | 3 | 21 | 6.8e-03 |
|  | small molecule biosynthetic process | 10 | 146 | 6.3e-04 |
|  | carboxylic acid biosynthetic process | 5 | 57 | 6.2e-03 |
|  | carbohydrate metabolic process | 9 | 178 | 1.0e-02 |
|  | hexose metabolic process | 4 | 61 | 4.1e-02 |
|  | cellular amino acid metabolic process | 10 | 143 | 5.5e-04 |
|  | cellular aromatic compound metabolic process | 7 | 53 | 7.6e-05 |
|  | aromatic amino acid family metabolic process | 3 | 13 | 1.3e-03 |
|  | cellular amine metabolic process | 13 | 158 | 8.8e-06 |
|  | L-serine biosynthetic process | 1 | 5 | 4.0e-02 |
|  | serine family amino acid metabolic process | 2 | 18 | 4.2e-02 |
|  | amine biosynthetic process | 5 | 49 | 3.3e-03 |
|  | cellular nitrogen compound biosynthetic process | 12 | 151 | 3.2e-05 |
|  | fatty acid beta-oxidation | 4 | 8 | 3.1e-06 |
|  | carboxylic acid catabolic process | 7 | 32 | 1.5e-06 |
|  | oxidation-reduction process | 7 | 146 | 3.3e-02 |
|  | monocarboxylic acid catabolic process | 5 | 9 | 8.1e-08 |
|  | sulfur amino acid metabolic process | 3 | 9 | 3.0e-04 |
|  | sulfur compound metabolic process | 4 | 28 | 2.2e-03 |
|  | cellular metabolic compound salvage | 1 | 5 | 4.0e-02 |
|  | defense response | 6 | 83 | 7.1e-03 |
|  | response to hypoxia | 1 | 5 | 4.0e-02 |
|  | antibacterial humoral response | 2 | 15 | 2.7e-02 |
|  | coenzyme metabolic process | 6 | 85 | 8.0e-03 |
|  | coenzyme biosynthetic process | 3 | 33 | 2.9e-02 |
|  | pteridine-containing compound biosynthetic process | 3 | 14 | 1.8e-03 |
|  | ribonucleoside monophosphate biosynthetic process | 4 | 24 | 1.1e-03 |
|  | nucleoside metabolic process | 4 | 30 | 3.0e-03 |
|  | valine metabolic process | 3 | 5 | 1.6e-05 |
|  | pentose-phosphate shunt | 2 | 11 | 1.3e-02 |
|  | polyamine biosynthetic process | 3 | 5 | 1.6e-05 |
|  | spermidine metabolic process | 3 | 5 | 1.6e-05 |
|  | sex differentiation | 3 | 39 | 4.9e-02 |
|  | vitellogenesis | 3 | 11 | 7.0e-04 |
|  | cellular iron ion homeostasis | 2 | 12 | 1.6e-02 |
|  | iron ion transport | 2 | 12 | 1.6e-02 |
|  | specification of segmental identity, head | 3 | 11 | 7.0e-04 |
|  | aromatic amino acid family catabolic process | 2 | 5 | 1.3e-03 |
|  | eye pigmentation | 3 | 21 | 6.8e-03 |
|  | defense response to Gram-positive bacterium | 1 | 5 | 4.0e-02 |
|  | cysteine metabolic process | 1 | 5 | 4.0e-02 |
|  | compound eye pigmentation | 1 | 5 | 4.0e-02 |

^1^Number of transcripts that show significantly increased abundance that are associated (directly or indirectly) with the Gene Ontology term

^2^Number of probesets present on the microarray that are associated (directly or indirectly) with the Gene Ontology term
