# Supplementary material for: Transcriptome Profiling of Sexual Maturation and Mating in the Mediterranean Fruit Fly, Ceratitis capitata
Source: PLoS One. 2012 Jan 27;7(1):e30857. doi: 10.1371/journal.pone.0030857 (PMC3267753; doi:10.1371/journal.pone.0030857)
Supplement: Table S6 — Significantly enriched biological process gene ontology annotations among transcripts that showed changes in abundance in mated female heads compared to mature virgin female heads. (DOC) [file pone.0030857.s007.doc]

Supplementary Table 6: Significantly enriched biological process gene ontology annotations among transcripts that showed changes in abundance in mated females compared to mature virgin females

| **Expression** | **Gene Ontology Term** | **Significant^1^** | **Annotated^2^** | **FDR-adjusted P-value** |
| --- | --- | --- | --- | --- |
| Lower abundance in mated female | **-** | - | - | - |
| Enriched in mated female | iron ion transport | 1 | 12 | 1.2e-02 |
|  | cellular iron ion homeostasis | 1 | 12 | 1.2e-02 |
|  | sensory perception of chemical stimulus | 1 | 37 | 4.4e-02 |
|  | peptidyl-amino acid modification | 1 | 25 | 3.0e-02 |
|  | mRNA cleavage | 1 | 14 | 1.5e-02 |
|  | periodic partitioning | 1 | 16 | 1.8e-02 |
|  | regulation of protein localization | 2 | 34 | 4.0e-03 |
|  | positive regulation of NFAT protein import into nucleus | 1 | 17 | 1.9e-02 |
|  | germ-band extension | 1 | 9 | 8.8e-03 |
|  | regulation of protein phosphorylation | 1 | 29 | 3.4e-02 |
|  | negative regulation of protein modification process | 1 | 5 | 4.0e-03 |
|  | negative regulation of phosphorylation | 1 | 6 | 5.5e-03 |

^1^Number of transcripts that show significantly increased abundance that are associated (directly or indirectly) with the Gene Ontology term

^2^Number of probesets present on the microarray that are associated (directly or indirectly) with the Gene Ontology term
