# Supplementary material for: Transcriptome Profiling of Sexual Maturation and Mating in the Mediterranean Fruit Fly, Ceratitis capitata
Source: PLoS One. 2012 Jan 27;7(1):e30857. doi: 10.1371/journal.pone.0030857 (PMC3267753; doi:10.1371/journal.pone.0030857)
Supplement: Table S7 — Transcripts that change in abundance in mated males with respect to mature virgin males. (DOC) [file pone.0030857.s008.doc]

**Supplementary Table 7: Transcripts that change in abundance in mated males with respect to mature virgin males. Up and down arrows refer to an increase or a decrease in transcript abundance in the mated males compared to virgin males. Unless otherwise stated BLASTX hits and the associated e-values refer to *Drosophila melanogaster* sequences**

| **GO category** | **Transcript** | **fold-change** | **Best BLASTX hit** | ***e*-Value** |
| --- | --- | --- | --- | --- |
| Reproduction | HC2667 | 2.04 ↓ | *Basigin* (*Bsg*) | 1e-57 |
|  | HS1072 | 2.01 ↓ | *loquacious* (*loqs*) | 1e-94 |
|  | HC1905 | 1.88 ↓ | *transforming acidic coiled-coil protein* (*tacc*) | 6e-35 |
|  | FS1820 | 2.59 ↑ | *Protein tyrosine phosphatase 61F* (*Ptp61F*) | 8e-69 |
| Behaviour | FS2804 | 2.01 ↓ | *CDP diglyceride synthetase* (*CdsA*) | 3e-93 |
|  | FC99 | 2.18 ↑ | *derailed* (*drl*) | 3e-17 |
|  | FS1943 | 1.81 ↑ | *double-time* (*dbt*) | 6e-16 |
| Chemoreception | HS3757 | 2.26 ↑ | *Odorant-binding protein 19d* (*Obp19d*) | 4e-12 |
| Immune system | HS3705 | 1.78 ↓ | *serpin-27A* (*Spn27A*) | 1e-56 |
|  | FC153 | 1.83 ↑ | *mutagen-sensitive 209* (*mus209*) | 9e-86 |
|  | HS283 | 2.73 ↑ | *myospheroid* (*mys*) | 2e-11 |
