# Supplementary material for: Transcriptome Profiling of Sexual Maturation and Mating in the Mediterranean Fruit Fly, Ceratitis capitata
Source: PLoS One. 2012 Jan 27;7(1):e30857. doi: 10.1371/journal.pone.0030857 (PMC3267753; doi:10.1371/journal.pone.0030857)
Supplement: Table S8 — Significantly enriched biological process gene ontology annotations among transcripts that showed changes in abundance in mated male heads compared to mature virgin male heads. (DOC) [file pone.0030857.s009.doc]

Supplementary Table 8: Significantly enriched biological process gene ontology annotations among transcripts that showed changes in abundance in mated males compared to mature virgin males

| **Expression** | **Gene Ontology Term** | **Significant^1^** | **Annotated^2^** | **FDR-adjusted P-value** |
| --- | --- | --- | --- | --- |
| Lower abundance in mated males | chitin catabolic process | 4 | 13 | 3.9e-04 |
|  | melanin metabolic process | 2 | 7 | 3.3e-02 |
| Enriched in mated males | imaginal disc-derived appendage development | 5 | 132 | 2.9e-02 |
|  | regulation of biological process | 19 | 1370 | 4.0e-02 |
|  | regulation of cellular process | 18 | 1254 | 3.8e-02 |
|  | biological regulation | 21 | 1538 | 3.2e-02 |
|  | regulation of MAP kinase activity | 1 | 6 | 4.8e-02 |
|  | negative regulation of protein serine/threonine kinase activity | 1 | 5 | 3.8e-02 |
|  | transition metal ion transport | 2 | 14 | 1.6e-02 |
|  | muscle attachment | 2 | 25 | 4.8e-02 |
|  | cell-matrix adhesion | 1 | 5 | 3.8e-02 |
|  | axonogenesis | 5 | 147 | 3.8e-02 |
|  | maintenance of epithelial integrity, open tracheal system | 2 | 9 | 7.8e-03 |
|  | anatomical structure homeostasis | 3 | 23 | 7.1e-03 |
|  | substrate-dependent cell migration, cell extension | 1 | 5 | 3.8e-02 |
|  | signal peptide processing | 1 | 5 | 3.8e-02 |
|  | negative regulation of cell size | 1 | 6 | 4.8e-02 |
|  | mismatch repair | 1 | 5 | 3.8e-02 |
|  | nucleotide-excision repair | 2 | 16 | 2.0e-02 |
|  | negative regulation of protein modification process | 1 | 5 | 3.8e-02 |
|  | negative regulation of phosphorylation | 1 | 6 | 4.8e-02 |
|  | regulation of protein localization | 3 | 34 | 1.2e-02 |
|  | response to metal ion | 1 | 6 | 4.8e-02 |
|  | behavioral response to cocaine | 1 | 6 | 4.8e-02 |

^1^Number of transcripts that show significantly increased abundance that are associated (directly or indirectly) with the Gene Ontology term

^2^Number of probesets present on the microarray that are associated (directly or indirectly) with the Gene Ontology term
