# Supplementary material for: Transcriptome Profiling of Sexual Maturation and Mating in the Mediterranean Fruit Fly, Ceratitis capitata
Source: PLoS One. 2012 Jan 27;7(1):e30857. doi: 10.1371/journal.pone.0030857 (PMC3267753; doi:10.1371/journal.pone.0030857)
Supplement: Table S9 — Correlation of microarray changes of transcript abundance with real-time qRT-PCR for 12 genes (log2 transformed fold expression). (DOC) [file pone.0030857.s010.doc]

**Supplementary Table 9: Correlation of microarray changes of transcript abundance with real-time qRT-PCR for 12 genes (log_2_ transformed fold expression)**

|  |  | **1d virgin vs. 4d virgin female** | | **4d mated vs. 4d virgin female** | | **1d virgin vs. 4d virgin male** | | **4d mated vs. 4d virgin male** | | **Pearson correlation** | |
| --- | --- | --- | --- | --- | --- | --- | --- | --- | --- | --- | --- |
| **Sequence** | **Gene** | **Arrays ± SE** | **qRT ± SE** | **Arrays ± SE** | **qRT ± SE** | **Arrays ± SE** | **qRT ± SE** | **Arrays ± SE** | **qRT ± SE** | **r** | **p** |
| HC321 | *takeout* | -0.193 ± 0.145 | 0.569 ± 0.091 | -0.054 ± 0.070 | -0.764 ± 0.104 | -0.578 ± 0.025 | 0.007 ± 0.147 | 0.021 ± 0.093 | -0.056 ± 0.116 | -0.281 | 0.719 |
| FS1844 | *smi35A* | 2.359 ± 0.159 | 3.231 ± 0.091 | 0.402 ± 0.091 | -0.222 ± 0.100 | 2.030 ± 0.188 | 2.130 ± 0.105 | 0.221 ± 0.119 | -0.570 ± 0.201 | 0.992 | 0.008 |
| HC2068 | *MSSPγ1* | 2.700 ± 0.287 | 5.700 ± 0.086 | 0.651 ± 0.399 | 0.411 ± 0.066 | N/A | 0.123 ± 0.125 | 0.266 ± 0.164 | 0.186 ± 0.073 | 0.994 | 0.071 |
| HC1570 | *Obp28a* | -0.498 ± 0.052 | 0.165 ± 0.234 | -0.179 ± 0.074 | -1.256 ± 0.128 | -0.419 ± 0.058 | 0.529 ± 0.234 | -0.240 ± 0.074 | -0.715 ± 0.273 | -0.915 | 0.085 |
| HC1629 | *Obp69a* | -0.465 ± 0.163 | -1.043 ± 0.325 | 0.093 ± 0.051 | -0.697 ± 0.070 | -0.710 ± 0.150 | -0.588 ± 0.307 | 0.560 ± 0.214 | 0.067 ± 0.098 | 0.731 | 0.269 |
| HC2265 | *Obp19d* | -1.296 ± 0.072 | -1.493 ± 0.280 | -0.202 ± 0.110 | -0.727 ± 0.240 | -0.497 ± 0.025 | -0.435 ± 0.299 | 0.151 ± 0.063 | 0.140 ± 0.262 | 0.923 | 0.077 |
| HC2536 | *Obp83a* | -1.135 ± 0.066 | -0.898 ± 0.277 | -0.197 ± 0.046 | -0.226 ± 0.086 | -0.871 ± 0.067 | -0.791 ± 0.284 | 0.108 ± 0.089 | 0.050 ± 0.160 | 0.995 | 0.005 |
| HS3757 | *Obp19d* | 1.032 ± 0.127 | 1.413 ± 0.261 | 0.839 ± 0.209 | -0.196 ± 0.103 | 1.355 ± 0.170 | 1.743 ± 0.323 | 0.851 ± 0.099 | 0.492 ± 0.136 | 0.873 | 0.128 |
| HC1181 | *Defensin* | -1.434 ± 0.327 | -1.921 ± 0.190 | 0.005 ± 0.155 | -0.382 ± 0.102 | -1.128 ± 0.133 | -1.899 ± 0.090 | 0.536 ± 0.052 | 0.134 ± 0.119 | 0.991 | 0.009 |
| FC1457 | *Relish* | 0.808 ± 0.030 | 0.363 ± 0.135 | -0.174 ± 0.044 | -0.370 ± 0.171 | -0.058 ± 0.083 | 0.059 ± 0.123 | -0.080 ± 0.125 | -0.378 ± 0.144 | 0.858 | 0.142 |
| FS1820 | *Ptp61F* | 0.907 ± 0.196 | 0.181 ± 0.405 | 1.095 ± 0.141 | 0.020 ± 0.092 | 0.385 ± 0.035 | -0.574 ± 0.261 | 1.437 ± 0.245 | -0.544 ± 0.082 | 0.118 | 0.882 |
| HC731 | *PGRP-LC* | 0.158 ± 0.061 | 0.225 ± 0.179 | 0.181 ± 0.063 | -0.420 ± 0.243 | -0.168 ± 0.092 | -0.871 ± 0.174 | -0.063 ± 0.062 | -0.762 ± 0.134 | 0.806 | 0.194 |
|  |  |  |  |  |  |  |  |  |  | 0.816 | <0.001 |

N/A indicates the absence of microarray or qRT-PCR data.
